# Distribution of lifespan gain from primary prevention intervention

**DOI:** 10.1136/openhrt-2015-000343

**Published:** 2016-03-11

**Authors:** Judith A Finegold, Matthew J Shun-Shin, Graham D Cole, Saman Zaman, Annette Maznyczka, Sameer Zaman, Rasha Al-Lamee, Siqin Ye, Darrel P Francis

**Affiliations:** 1 International Centre for Circulatory Health, National Heart and Lung Institute, London, UK; 2 Newcastle upon Tyne Hospitals NHS Foundation Trust, Newcastle, UK; 3 Department of Medicine, Center for Behavioral Cardiovascular Health, New York, New York, USA

**Keywords:** LIPIDS

## Abstract

**Objective:**

When advising patients about possible initiation of primary prevention treatment, clinicians currently do not have information on expected impact on lifespan, nor how much this increment differs between individuals.

**Methods:**

First, UK cardiovascular and non-cardiovascular mortality data were used to calculate the mean lifespan gain from an intervention (such as a statin) that reduces cardiovascular mortality by 30%. Second, a new method was developed to calculate the probability *distribution* of lifespan gain. Third, we performed a survey in three UK cities on 11 days between May–June 2014 involving 396 participants (mean age 40 years, 55% male) to assess how individuals evaluate potential benefit from primary prevention therapies.

**Results:**

Among numerous identical patients, the lifespan gain, from an intervention that reduces cardiovascular mortality by 30%, is concentrated within an unpredictable minority. For example, men aged 50 years with national average cardiovascular risk have mean lifespan gain of 7 months. However, 93% of these identical individuals gain no lifespan, while the remaining 7% gain a mean of 99 months. Many survey respondents preferred a *chance* of large lifespan gain to the equivalent life expectancy gain given as certainty. Indeed, 33% preferred a 2% probability of 10 years to fivefold more gain, expressed as certainty of 1 year.

**Conclusions:**

People who gain lifespan from preventative therapy gain far more than the average for their risk stratum, even if perfectly defined. This may be important in patient decision-making. Looking beyond mortality reduction alone from preventative therapy, the benefits are likely to be even larger.

**Key questions**

**What is already known about this subject?**

- Current clinical practice examines cardiovascular risk over fixed time windows that are typically much shorter than a healthy individual's life expectancy. Therefore, when advising patients about possible initiation of primary prevention treatment, clinicians currently do not have information on expected impact on lifespan, nor how much this differs between individuals.

**What does this study add?**

- Our study shows that the probability distribution of expected benefit from primary prevention therapy for individuals starting from an identical baseline is far from uniform, with people who gain lifespan from preventative therapy gaining far more than the average for their risk stratum. In addition, the spectrum of lifespan gain has a similar range between low-risk and high-risk patients: the difference is not in the size of lifespan in those that benefit but in the proportion of patients who benefit.

**How might this impact on clinical practice?**

- The results of this study suggest an opportunity to broaden prescription of primary prevention, since it suggests that younger patients, despite having lower initial estimated cardiovascular risk, may be in a position to gain the most from extended therapy.

## Introduction

Deciding whether or not to start preventative therapy can be challenging. Current guidelines recommend a shared decision-making process, beginning with estimation of cardiovascular risk.^1^ ^2^ However, when calculated over a lifetime (the usual intended duration of preventative medication), cardiovascular risk turns out to be high in everyone.^3^ ^4^ Therefore, perhaps in order to spread people along a spectrum, cardiovascular risk is commonly assessed over a fixed time window such as 10 years.

A challenge commonly raised by opponents of primary prevention is that many patients given preventative medication could be argued to not ‘need’ it because, even without treatment, they will not experience a cardiovascular event.^5^ Clinicians understand that averaging benefit over a population is a necessary simplification, as whenever there is one patient who gains less than average from an intervention, there is another who has an identical risk factor profile at baseline but gains more.^6–8^ Discussing this uncertainty over an individual's future gain is rare, perhaps because information on it is not currently available in a manner that can easily be conveyed to patients.^9^ ^10^ In principle, this could be described as a distribution of different sizes of lifespan gain among individuals who at the outset are indistinguishable.

To address these gaps in knowledge, we performed a three-part study. First, we used published cardiovascular and non-cardiovascular mortality data to calculate the mean lifespan gain from primary prevention interventions such as a statin. Second, we used a simulation approach to calculate a probabilistic distribution of lifespan gain at the individual level for patients who at baseline have identical cardiovascular risk profiles. Finally, we carried out a survey to assess empirically how people perceive benefit gains from primary prevention therapy when these benefits are described in fixed or probabilistic terms.

## Methods

### Calculation of mean lifespan gain

Age-specific cardiovascular mortality data and established hazard ratios achievable by preventative therapy were used to calculate the expected, or mean, lifespan gain for men and women with different levels of baseline cardiovascular risk using standard multiple decrement life-table methods.^11^ Baseline life expectancy was calculated using published age-specific mortality data in England and Wales in 2012^12^ and population data^13^ obtained from the UK Office for National Statistics (ONS). Deaths from ischaemic heart disease (ICD-10 codes I20–I25) and cerebrovascular disease^14^ (I60–I69) were combined to calculate age-specific cardiovascular mortality.

Data on the national average mean, and the distribution of blood pressure (BP), smoking status and cholesterol were obtained from the QRESEARCH database (2005).^15^ Separate life expectancy values were calculated for each combination of cardiovascular risk factors (smoking, systolic BP, total cholesterol, age and sex) using the SCORE algorithm.^16^ We have not included separate life-tables for patients with diabetes because they routinely receive primary prevention.^2^

Reduction in cardiovascular mortality was calculated for a single-agent preventative therapy—for example, a statin that has been shown in meta-analyses to reduce cardiovascular death by 20–30%.^17^ We defined the average expected longevity benefit as the difference between baseline life expectancy and life expectancy with intervention that reduces cardiovascular (but not non-cardiovascular) mortality by 30%. We covered ages of initiation of preventative therapy from 50 years upwards.

### Distribution of lifespan gain among individuals within the same risk stratum

#### Motivation and outline of calculation method

Even within a group of people with identical cardiovascular risk, individuals will each have different lifespans and different individual gains in lifespan from prevention, because of the effect of chance. To calculate an individual gain, we need to quantify for each individual a pair of lifespans which use an identical play of chance but with different thresholds for a fatal event (see online supplementary appendix 1).

The outline of this process is most easily appreciated using an analogy. Imagine mortality being determined purely by throwing a pair of dice every day. If an individual throws a six on either of their dice, their life ends. Running this for many days permits a lifespan to be calculated. The same dice throws can then be re-evaluated to deliver reduced mortality risk but identical play of chance. For example, if a double six was now required for a fatal event, then many of the throws that had been considered fatal would now not be fatal, so lifespan would likely be longer. This ability to evaluate in a single simulated individual (ie, an identical play of chance) the impact of a risk reduction on lifespan is unique to this approach. This cannot be established from clinical trials because each patient lives only once.

This process can be carried out for multiple simulated individuals at identical baseline risk. Each individual has their own unique single set of dice throws for which two lifespans are calculated. Conducting this process for thousands of simulated individuals who are identical at the outset allows us to state what proportion of them would gain lifespan from the intervention and by how much. The existence of a distribution of lifespan gain should not be misunderstood to reflect variation in risk between individuals: all had identical risk and the differences result entirely from the play of chance.

#### Details of formal calculation method

We wrote software in Matlab to carry out these calculations. For purposes of replication, we wrote the same algorithm in Python to confirm identical results. Online supplementary Appendix 1 shows software code in both languages with a full explanation of the method of calculation, which is a Monte Carlo simulation with duplicate evaluation of identical stochastic data.

### Conveying life expectancy gains to patients: survey of general public to assess preference

We tested members of the general public for their preference between a certainty of a small gain in healthy lifespan (1 year) versus a percentage chance of a larger gain in healthy lifespan (10 years). Adults were approached in public thoroughfares in three different multiethnic cities in the UK (London, Leicester and Newcastle) on 11 separate days and invited to participate in a brief verbal survey. There were no inclusion or exclusion criteria.

Respondents were randomly allocated to one of five versions of the survey in which the percentage chances of the larger gain are 2%, 5%, 10%, 20% and 50% (see online supplementary Appendix 2). Each respondent answered the question for only one of these five different comparisons. Each comparison was to be answered by at least 55 respondents, which would provide a precision (95% CI) of±13%.

This survey did not require ethical committee approval, because it assessed attitudes to an explicitly imaginary intervention and was conducted with members of the general public without collection of personally identifiable information. This principle has been established by discussion with our local ethical committee.^18^

#### Sample size calculation for survey

We calculated the necessary sample size based on achieving a target level of precision. We wanted to quantify the proportion of respondents preferring each of the two choices with a precision (95% CI) of ±5%. This required 385 respondents. We aimed to sample for complete days until the count exceeded 385 respondents.^19^

## Results

### Life expectancy gain from a lifetime of preventative therapy

**Figure 1:**
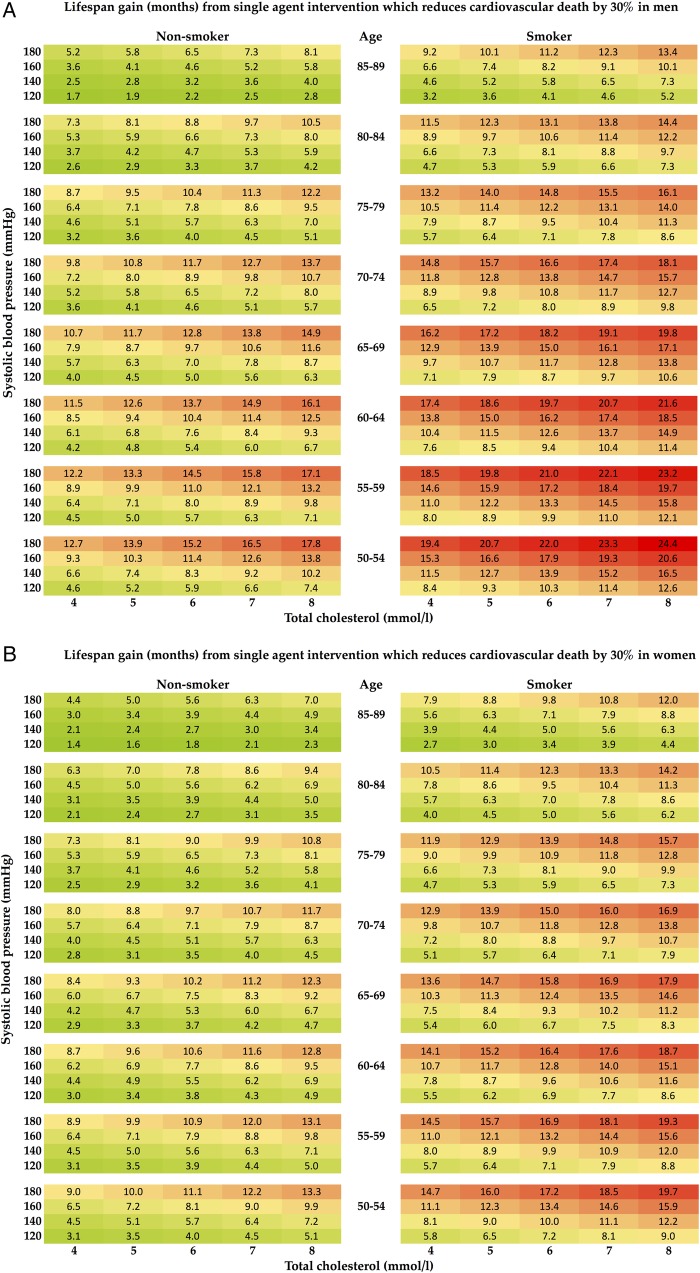
Tables showing mean lifespan gain in months for men (A) and women (B) obtained from a single-agent preventative therapy with CV risk reduction of 30%. Lifespan gain is calculated from the patient's age, sex, blood pressure, cholesterol level and smoking status. CV, cardiovascular.

**Figure 2:**
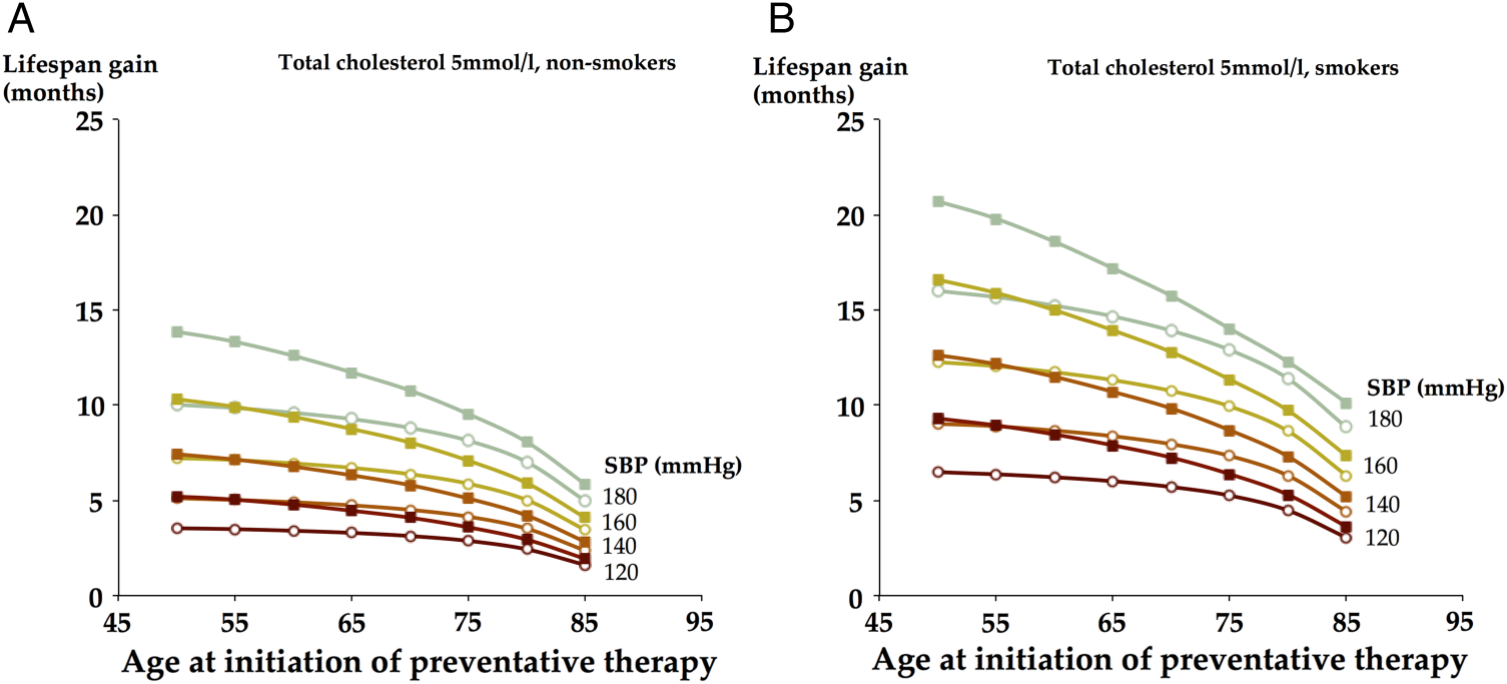
For any combination of cardiovascular risk factors, the lifespan gain from initiation of intervention decreases with increasing age of initiation. Change in lifespan gain as age of initiation of therapy increases with differing baseline cardiovascular risk in non-smokers (A) and smokers (B) taking a daily single-agent therapy—for example, a statin with 30% cardiovascular risk reduction. Men are shown with square, filled data points, women with circular, unfilled data points.

From the mean lifespan gain for any combination of baseline risk factors (figure 1), the effect of the age of initiation of therapy on mean lifespan gain can be seen (figure 2). For example, among non-smokers starting preventative therapy at the age 50, the life expectancy gain ranges from 3.1 months for women in the lowest risk stratum (total cholesterol 4 mmol/L, SBP 120 mm Hg) to 17.8 months for men in the highest risk stratum (total cholesterol 8 mmol/L, SBP 180 mm Hg).

It is notable that although risk, and absolute risk reduction from intervention, increases with age, this does not translate into mean lifespan gain increasing with age of initiation of intervention. In fact, for any combination of cardiovascular risk factors, the potential lifespan gain from initiation of intervention decreases with increasing age of initiation. The gain for initiation at age 50 is approximately twofold to threefold larger than the gain for initiation at age 80.

### Distribution of lifespan gain within a primary prevention population compared with mean lifespan gain

**Figure 3:**
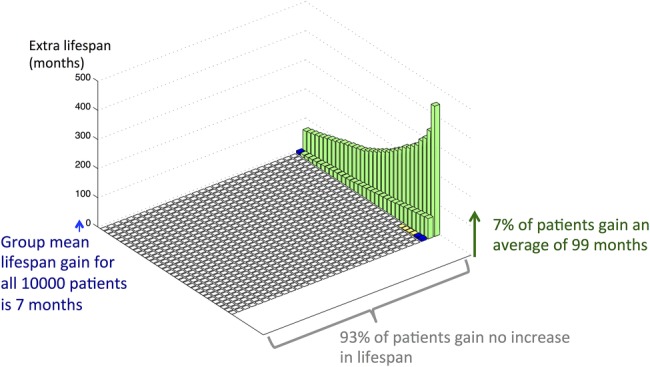
Distribution of lifespan for men initiating preventative therapy (with a risk reduction of 30%) at the age of 50, with national average blood pressure and national average cholesterol. Each small square indicates one representative individual taking a lifetime of preventative therapy. For the individuals whose lifespans are longer with medication, a bar is drawn whose height represents their individual lifespan gain from medication: white columns represent individuals who gain no extra lifespan, yellow columns represent individuals who gain lifespan less than the group mean gain, blue columns represent individuals who gain an amount of lifespan similar to the group mean gain and green columns represent individuals whose gain is more than the group mean gain. The graph makes it clear that although only a minority of the cohort (7%) gain any lifespan from a lifetime of preventative therapy; nevertheless, this relatively small group gain is much more (99 months) than the group mean gain (7 months).

A group of individuals starting preventative therapy at the same age, even if their baseline characteristics are identical, will have a range of different individual lifespan gains. The distribution of lifespan gain from taking daily preventative therapy with a risk reduction of 30% is shown in figure 3 for men with national average cardiovascular risk starting preventative therapy at the age of 50. Notably, the great majority gain no lifespan, while the minority that do gain, gain much more than the group average increase in lifespan. For example, for a 50-year-old, non-smoker, non-diabetic man with average cholesterol and BP, mean life expectancy gain is 7 months starting preventative therapy. However, among such individuals, 93% gain no extra lifespan, but the remaining 7% gain an average of 99 months.

### Impact of cardiovascular risk on the distribution of lifespan gain within a risk stratum

The distribution of lifespan gain is dependent on baseline cardiovascular risk. For a group of lower risk individuals, for example, women with half-national average baseline cardiovascular risk for women, mean life expectancy gain from initiating therapy at age 50 is 3 months. This arises from a 3.4% subset of patients who, between them, gain an average of 92 months, while the remaining 96.6% do not gain any increase in life expectancy.

**Figure 4:**
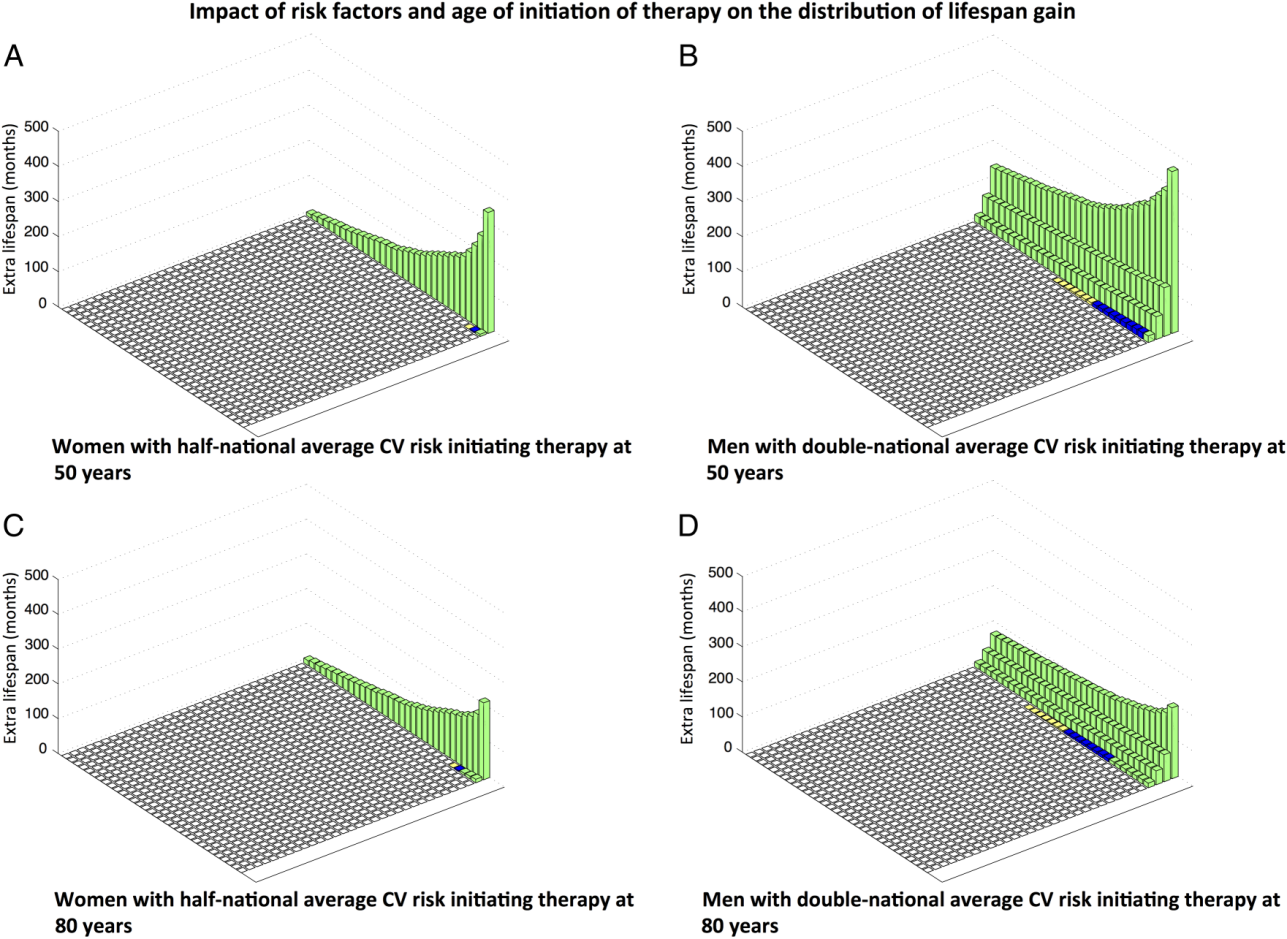
Impact of risk factors and age of initiation of therapy on the distribution of lifespan gain. ‘National average risk’ refers to a cohort beginning at age 50, and each year experiencing the national average cardiovascular risk that rises with age (age-specific mortality data obtained from the UK ONS). (A) Women with half-national average CV risk initiating therapy at 50 years. Mean life expectancy gain 3 months, 96.6% gain no extra lifespan, but the remaining 3.4% gain an average of 92 months. (B) Men with double-national average CV risk initiating therapy at 50 years. Mean life expectancy gain 12 months, 88.9% gain no extra lifespan, but the remaining 11.1% gain an average of 107 months. (C) Women with half-national average CV risk initiating therapy at 80 years. Mean life expectancy gain 2.5 months, 96.4% gain no extra lifespan, but the remaining 3.6% gain an average of 70 months. (D) Men with double-national average CV risk initiating therapy at 80 years. Mean life expectancy gain 6 months, 88.5% gain no extra lifespan, but the remaining 11.5% gain an average of 56 months. CV, cardiovascular; UK ONS, UK Office for National Statistics.

In a higher risk group, for example, males initiating therapy at 50 years with double the national average baseline risk for men, the mean life expectancy gain is higher (12 months). This is composed of a more substantial subset (11.1%) of patients who gain an increase in lifespan of average 107 months (figure 4).

Thus, those that gain lifespan in the high-risk population gain a very similar amount to those that gain lifespan in the low-risk population. What differs greatly between the populations is the proportion who benefit, which is approximately three times larger for the high-risk group in the example above.

### Sensitivity analysis

We explored this pattern in a sensitivity analysis, which examined alternate scenarios (table 1). We covered combinations of proportions of cardiovascular risk (15%, 20%, 25%) and hazard reductions from intervention (0.2, 0.3, 0.4). The pattern of results was that higher proportions of cardiovascular risk and larger hazard reductions gave higher mean lifespan gains, and this was composed of a larger proportion of patients benefitting but almost no change in the mean lifespan gain among those who gained lifespan.

**Table 1:** Sensitivity analysis examining alternate scenarios

	Male				Female			
	Mean lifespan gain (months)	Proportion not benefitting (%)	Proportion that benefits (%)	Mean lifespan gain for those benefitting (months)	Mean lifespan gain (months)	Proportion not benefitting (%)	Proportion that benefits (%)	Mean lifespan gain for those benefitting (months)
15% cardiovascular mortality								
Hazard reduction 0.2	3.0	97.1	2.9	103.4	2.9	97.1	2.9	97.4
Hazard reduction 0.3	4.6	95.6	4.4	105.0	4.1	95.8	4.2	97.8
Hazard reduction 0.4	6.1	94.1	5.9	103.7	5.9	94.1	5.9	100.6
20% cardiovascular mortality								
Hazard reduction 0.2	4.2	96.0	4.0	104.3	3.7	96.2	3.8	96.6
Hazard reduction 0.3	6.1	94.0	6.0	102.3	5.9	94.1	5.9	99.5
Hazard reduction 0.4	8.1	92.1	7.9	102.5	8.0	92.3	7.7	103.9
25% cardiovascular mortality								
Hazard reduction 0.2	5.2	94.9	5.1	101.1	4.7	95.2	4.8	97.4
Hazard reduction 0.3	7.7	92.5	7.5	102.3	7.5	92.7	7.3	103.8
Hazard reduction 0.4	10.5	90.1	9.9	106.4	10.5	90.3	9.7	108.2

### Impact of delayed initiation on lifespan gain

On first inspection of figure 2, it may appear that there is little to gain from starting intervention at the youngest age of initiation because lifespan gain is not falling rapidly with age. Our analysis permits the same patient's life course to be recalculated without intervention or with intervention started at different ages. This permits the extra lifespan gain in that individual from earlier or later initiation of therapy to be directly evaluated. Figure 4 reveals that with increasing age, the percentage of patients that benefit stays relatively stable, but the mean possible lifespan gain and the lifespan gain in those that benefit decrease.

For example, in men initiating therapy at age 50, mean lifespan gain available is 12 months with 11.1% gaining an average of 107 months. If this same group of identical individuals started therapy at 80 years instead, the mean lifespan gain is reduced to 6 months, which arises from the percentage benefitting staying relatively constant at 11.5% but those benefitting gaining a far smaller amount (56 months).

### Survey of general public's preference between certainty of small lifespan gain and chance of large lifespan gain

Discussing life expectancy gains with patients tacitly relies on the principle that they would consider it equally desirable to gain a 50% chance of two extra life-years or a certainty of 1 year. We assessed how individuals evaluate potential benefit of primary prevention therapy when such benefits were presented in fixed or probabilistic terms. Between May and June 2014, 396 participants were recruited after approaching 465 members of the public. Their mean age was 40 years (SD 17 years), 55% were male and 4% had had myocardial infarction or stroke (see online supplementary Appendix 3).

**Figure 5:**
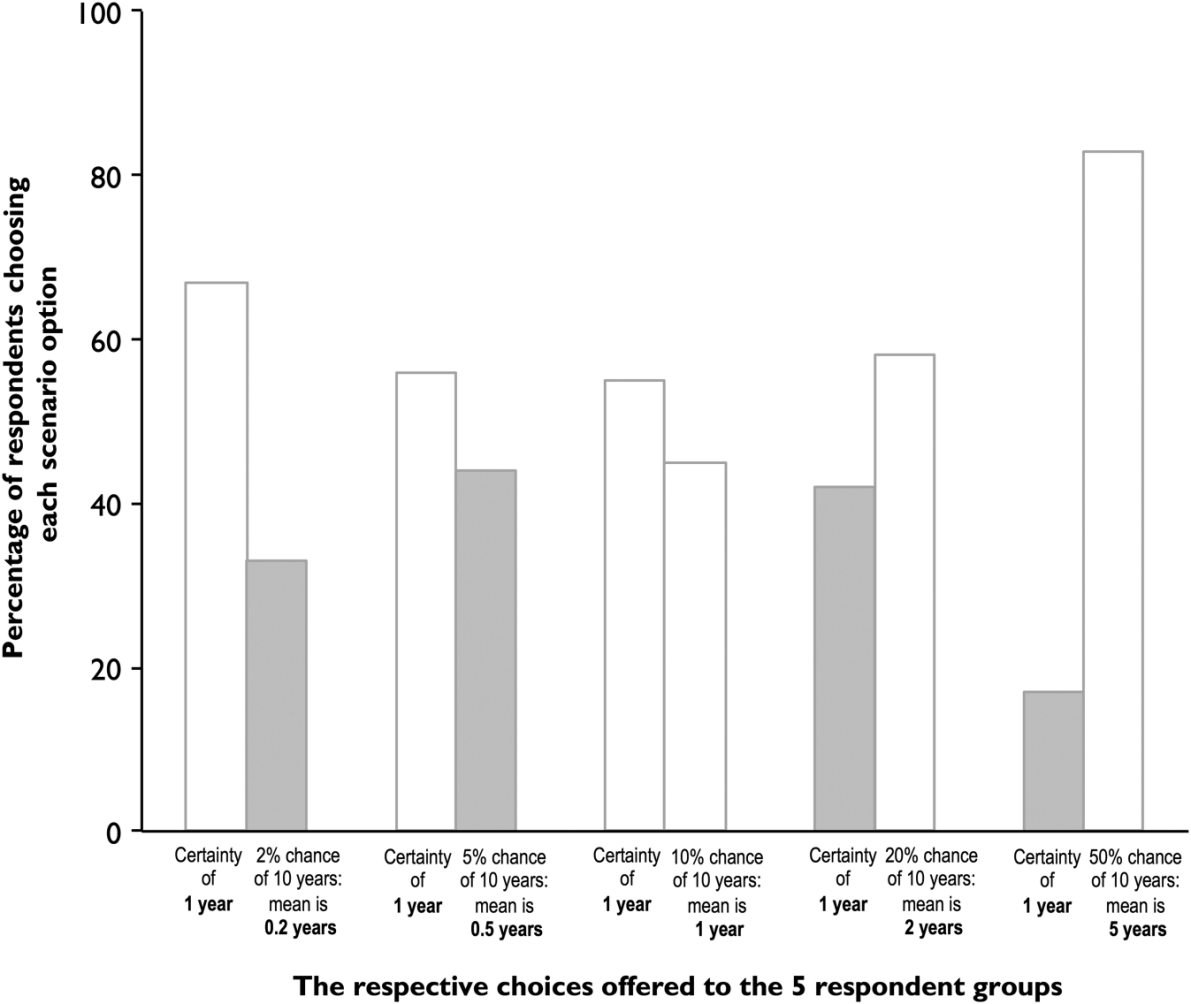
Patients’ preferences do not always match maximisation of lifespan. Respondents were randomly allocated to one of five groups. Each respondent chose between the certainty of 1 year of lifespan gain and a % chance of 10 years of lifespan gain. The different % chances offered to the five groups were 2%, 5%, 10%, 20% and 50%, respectively, equivalent to a mean life expectancy increase of 0.2, 0.5, 1, 2 and 5 years. The bars show the proportion of respondents in each group choosing the certainty option or the % chance option. We have shaded in grey the respondents who were choosing an option with lower mean life expectancy. As the probability offered increased, progressively more respondents chose the % chance option. Nevertheless, at each offered probability, many respondents preferred the option that gave the lower mean life expectancy (grey columns).

The findings are shown in figure 5. Some respondents (left pair of bars) were asked to choose between the certainty of 1 year of lifespan gain and a 2% chance of 10 years of lifespan gain which is equivalent to a life expectancy increase of 0.2 years. For other respondents, the probability offered was 5%, 10%, etc. For the respondents offered 2% or 5% probability as the chance option, choosing the chance option misses substantial opportunity for life expectancy gain, as shown by grey shading of columns. Conversely, for respondents offered 20% or 50% probability as the chance option, choosing the certainty option misses an even larger opportunity for lifespan gain, again shown by grey shading of columns. As the probability offered increased, progressively more respondents chose the chance option. Nevertheless, at each offered probability, many respondents preferred the option that gave the shorter life expectancy.

## Discussion

Current clinical practice examines risk over fixed time windows that are typically much shorter than a healthy individual's life expectancy. Our present analysis, which addresses entire lifespan, shows that for any cardiovascular risk factor profile, it is the younger individuals who gain the most lifespan from initiation of primary prevention therapy.

Second, our results show that although the great majority gain no lifespan, the minority that do gain, gain much more than the group average increase in lifespan. The spectrum of lifespan gain has a similar range between low-risk and high-risk patients: the difference is not in the size of lifespan in those that benefit but in the proportion of patients who benefit.

Third, our survey indicates that, when presented with probabilistic information, many individuals have a personal preference for certainty of a small gain or for a chance of a large gain, not corresponding to which of those is mathematically larger. This suggests that when discussing benefit of preventative therapy with patients, we might present lifespan gain both ways.

### More gain despite lower risk? The age paradox

When discussing whether to initiate primary prevention therapy, clinicians commonly counsel patients using metrics such as estimated cardiovascular risk over a fixed time window—for example, percentage risk of cardiovascular disease over 10 years.^1^ However, treatment is not intended for a fixed time window but for life. Although older individuals tend to have higher estimated risk, it is likely that initiation of primary prevention therapy in younger individuals, whose estimated risk is lower but who meet eligibility criteria, gives larger gains in lifespan. These results are based on the HR for cardiovascular event reduction calculated from meta-analysis of randomised controlled trials of statins. Our calculations use the Gompertz method to build in recognition that non-cardiovascular mortality (not improved by statins) will progressively rise with age, attenuating the overall HR for mortality from statins. However, if statins were to have a previously undescribed effect of increasing mortality after prolonged use, our calculations would not be valid. Few data with randomisation retained in the long term do seem to suggest a sustained hazard reduction while randomised to statin.^20^

A more complex issue is the impact of smoking. On the one hand, smokers have a higher risk of cardiovascular disease, which would tend to increase the gain from preventative therapy. On the other hand, smokers have a higher risk of death from non-cardiovascular disease, for example, cancer, which would tend to decrease the gain from preventative therapy. It is difficult to confidently quantify whether preventative therapy adds more or less extra lifespan in smokers than in non-smokers; therefore, our study refrains from attempting to do so. Likewise, our study addresses patients who have already been risk stratified in whatever manner is considered cost-effective. For example, this might be by demographic and physiological measurements, or by newer technology such as CT calcium scoring. Whatever the stratification process, there is no way to tell which individual among many with the same risk level will die first, or which individual will gain the most from preventative therapy. Our paper addresses the uncertainty that cannot be removed owing to cardiovascular mortality possessing substantial inherent unpredictability within individuals in the long term.

### Treat all versus tailored approach

Our study has implications for the debate between offering treatment to all or spending resources on a tailored approach for primary prevention. The lifetime risk of cardiovascular disease for a man with average characteristics is 37%.^21^ Some people may consider this a large value and worthy of preventative steps. Others may consider it too small. Our study indicates that individuals will vary in their opinions and it would be worth asking them their view individually.

Separately, modern technology may allow options beyond demographic and physiological measures to assess risk stratum. However, regardless of the method of risk evaluation, there will remain inherent uncertainty in the actual outcome for the individual. Our study addresses this irremovable uncertainty in individualised medical practice.

### Do people focus on average gain or maximum gain?

It is natural for healthcare provision planners to focus on average gain from an intervention, as mathematically this is the simplest summary. It has the desirable property of consistently increasing if the probabilities stay the same and the individual magnitudes increase, and also if the individual magnitudes stay the same and the probability of benefit increases. It is the basis for indices such as quality-adjusted life-years (QALYs) that are widely used in healthcare economic planning.^22^ Our study is not an economic analysis. It does not quantify dollar costs or attempt to attach dollar values to the benefit of having more life. This is because patients rarely decline primary prevention at the outset on the basis of too great financial cost to the healthcare system, nor too little financial gain from intervention. Instead, our analysis focuses on dimensions widely understood and brought up by patients—namely, magnitude of benefit and chance of receiving it.

People do not always choose the option offering the mathematically maximal average benefit.^23^ Our survey illustrates that people often prefer a small chance of a large benefit over the certainty of a small benefit, even when the mathematical average gain from the former is smaller. Similar phenomena are described in the fields of behavioural economics and cognitive psychology under the umbrella of prospect theory.^24^ Taken together, these insights have important implications for the shared decision-making process for considering primary prevention statin therapy.

### Study limitations

Our calculation of lifespan gain is based on the relative risk reduction for cardiovascular mortality from preventative medication being approximately the same across risk groups and across ages. In particular, we have relied on the observation from large-scale meta-analyses 25 of statins that there is no sign that patients with low-to-intermediate estimated cardiovascular risk have a weaker hazard reduction. However, if there is any particular risk stratum in which cardiovascular mortality is reduced to a different extent by medical intervention, then our results would need to be modified.

Our analysis pays no regard to the possibility of adverse effects of therapy. For primary prevention statins, for example, there is a documented increase of diagnosis of diabetes of approximately 0.5% which partly counteracts the 0.5% decrease in absolute mortality, 1% decrease in myocardial infarction and 0.3% decrease in strokes.^26^ In the longer term, it is conceivable that the higher rate of diabetes could increase cardiovascular event rates. However, even the longest randomised controlled trials have shown nothing other than continued reduction of cardiovascular event rates in the statin arms, to the end of the randomised period and beyond.^27^

We used contemporary mortality data for all age strata to construct the life-table, but it should be remembered that there has been a secular trend to falling mortality,^28^ which is likely to continue for many years to come. Therefore, when people currently aged 50 eventually reach the age of 80, they will be facing not the mortality of current 80-year-olds (used in our life-tables) but a future mortality which is likely to be lower. Thus, the actual life-years gained for people starting preventative therapy now will likely be larger than computed in our life-tables.

Life expectancy and cardiovascular and non-cardiovascular mortality rates vary between countries.^28^ Therefore, our life-tables, built from the UK data, may be slightly different in other geographies. We have previously provided full details of this method in an online supplementary appendix to a previous publication.^18^

We have used the SCORE system to assess cardiovascular risk as suggested in current European Society of Cardiology (ESC) guidelines. It is widely used in the UK since it has been present in the British National Formulary (equivalent to the American Physicians’ Desk Reference) since September 2000. Any risk score system can be criticised for not being representative of other populations that may differ because of geography, or because of secular changes that have occurred in the time between system development and current application. For example, in developed countries, age-specific death rates from cardiovascular disease are falling, partly from the widespread adoption of preventative steps, and only the increasing population age is causing the absolute numbers of cardiovascular deaths to remain constant. Nevertheless, in other countries, even age-specific death rates from cardiovascular disease are still in their rising phase. The principle of our study, however, is applicable to any risk score that shows a gradation of risk at each age and increasing risk with age.

In our survey, the participants are a convenience sample from several places in one country only, which limits its external validity. Moreover, it is possible that persons in public places may differ from the true general population in their relative preference between certainty of small benefit and chance of large benefit. We perceived that the advantages of high participation might outweigh the statistical imperfection of a convenience sampling strategy. To encourage high participation, we intentionally ensured that the survey was relatively short and easy to complete. This focus on high participation prevented any simultaneous assessment of numeracy or educational level; therefore, we do not know whether variation in numeracy in our survey participants could have influenced our results. Further research to confirm our findings might additionally address the potential impact of numeracy on probabilistic presentation of lifespan benefit.

Our survey age spectrum was deliberately wide because this study followed on from a previous study in which we recognised that the lifespan gain from choosing primary prevention is certainly no lower for younger patients than older.^18^ If we had focused entirely on older patients, we would have had less ability to explore variation in preference across this wide age spectrum.

Our model of the distribution of lifespan gain operates on the basis that there is a cohort of patients with identical baseline risk and whose risk rises only with age, with no other differentiating factors. A more complex model could alternatively be constructed which would include the counting of, for example, non-fatal events, which might produce additional variables such as extra lifespan free of cardiovascular events. While many non-fatal events leave no long-term disability, some cause long-term incapacitation by, for example, heart failure. Degrees of reduction of long-term morbidity from primary prevention are more difficult to quantify than those of mortality but are likely to be approximately parallel given the nature of the disease process. On this basis, if the benefit in morbidity and mortality could be calculated, it would likely be larger than described here and the proportion of people benefitting would be larger.

## Conclusions

Current practice is to advise patients whether to start primary prevention therapy based on their cardiovascular risk over a fixed time horizon. Our results suggest an opportunity to broaden this approach, since it suggests that younger patients, despite having lower initial estimated cardiovascular risk, may be in a position to gain the most from extended primary prevention therapy. Furthermore, our numerical analysis indicates that the probability distribution of expected benefit from primary prevention therapy for individuals starting from an identical baseline is far from uniform, consisting instead of many who gain no extra lifespan plus a small minority who gain a large extra lifespan gain (many times greater than the group mean life expectancy gain).
